# Ultraearly Hematoma Growth in Acute Spontaneous Intracerebral Hemorrhage Predicts Early and Long-Term Poor Clinical Outcomes: A Prospective, Observational Cohort Study

**DOI:** 10.3389/fneur.2021.747551

**Published:** 2021-12-15

**Authors:** Wen-Juan Wang, Jing-Jing Lu, Li-Ping Liu, Jiao-Kun Jia, Xing-Quan Zhao

**Affiliations:** Department of Neurology, Beijing Tiantan Hospital, Capital Medical University, Beijing, China

**Keywords:** intracerebral hemorrhage, ultraearly hematoma growth, outcome, predictors, CT

## Abstract

**Aims:** Although prognostic importance of ultraearly hematoma growth (uHG) in acute, non-traumatic intracerebral hemorrhage (ICH) has been established for early outcomes, longer-term clinical outcomes are lacking. We aimed to determine the association of uHG with early and 1-year clinical outcomes after acute ICH in a larger and broader range of patients.

**Methods:** We studied 589 patients with acute (<6 h) spontaneous ICH. uHG was defined as baseline ICH volume/onset-to-imaging time (OIT) (ml/h). Multivariable logistic regression analyses were performed to determine the association of uHG with in-hospital mortality, 90-day, and 1-year poor outcome [3 ≤ modified Rankin Scale (mRS)] after ICH.

**Results:** The median speed of uHG was 4.8 ml/h. uHG > 9.3 ml/h was independently related to in-hospital mortality [odds ratio (OR) 2.81, 95% CI 1.52–5.23], 90-day poor outcome (OR 3.34, 95% CI 1.87–5.95), and 1-year poor outcome (OR 3.59, 95% CI 2.01–6.40) after ICH. The sensitivity of uHG > 9.3 ml/h in the prediction of in-hospital mortality, 90-day poor outcome, and 1-year poor outcome was 68.8, 48.0, and 51.1%, respectively.

**Conclusions:** Ultraearly hematoma growth was a useful predictor of in-hospital mortality, 90-day, and 1-year poor outcome after acute ICH. The combination of both uHG and baseline ICH volume could allow better selection of patients with ICH at high risk of poorest clinical outcomes for future clinical trials to improve early- and long-term clinical outcomes.

## Introduction

The incidence and the case fatality of spontaneous intracerebral hemorrhage (ICH) have not decreased and only 12–39% of the survivors live independently 6 months post-ICH onset (1). Baseline hematoma volume has proven to be one of the most powerful predictors of poor outcome in patients with ICH (2, 3). Hematoma growth (HG), which occurs mainly during the first 6 h, has been shown to be an independent determinant of early deterioration, death, and disability in patients with ICH (4, 5). Due to the enlargement of hematoma in the first few hours, the impact of initial ICH volume on outcome may vary widely depending on the time from symptom onset to baseline CT scan [onset-to-imaging time (OIT)], even in patients within 6 h of onset (4–6). Therefore, the adjustment of initial ICH volume by OIT may improve the accuracy of baseline ICH volume to predict acute ICH clinical outcomes.

Ultraearly hematoma growth (uHG), calculated as the baseline hematoma volume (ml) divided by OIT (hours), has been proven to be a predictor of poor clinical outcomes in patients with acute ICH (6–8). However, these studies included a subpopulation of patients with ICH, which may, therefore, reduce its generalizability to clinical practice. Furthermore, the association of uHG with 1-year outcome after acute ICH has not been studied. The objectives of this study were to investigate the impact of uHG on early and 1-year clinical outcomes in a larger and broader range of patients with ICH. We also aimed to compare the ability of uHG and baseline (<6 h) ICH volume to predict ICH early and long-term clinical outcomes.

## Method

### Study Design and Population Eligibility

This study was a multicenter, prospective, observational cohort study. Thirteen hospitals in Beijing participated. From December 2014 to September 2016 patients who presented with an acute symptomatic and CT confirmed ICH were recruited to the study. Patients aged 18 years or older were eligible for entry if they presented within 6 h from symptoms onset with a spontaneous ICH. Exclusion criteria included those patients with uncertainty on the exact time from symptom onset, secondary cause of ICH (e.g., tumor, arteriovenous malformation, or rupture of aneurism), surgical hematoma evacuation or other neurosurgical intervention, prestroke-modified Rankin Scale (mRS) was >2 or lost to follow-up at 1 year.

The study protocol was approved by the Institutional Review Board (IRB) of the Beijing Tiantan Hospital affiliated with Capital Medical University. The ethics committee(s) approved consent by proxy in the ethics statement. Requirement for additional ethics approval differed among participating hospitals, and additional consent was obtained if required by the attending site ethics board. All patients who had the capacity of understanding and signing or their legally authorized representative gave written informed consent, according to the requirements established by each site ethics board. Participating centers collected data and submitted it online to the coordinating center of Beijing Tiantan Hospital, Capital Medical University.

### Procedures

Relevant demographic and clinical characteristics were recorded. The National Institutes of Health Stroke Scale (NIHSS) and Glasgow coma scale (GCS) on admission were collected. The time of onset was defined as the time of symptoms onset or the last time seen normal. Baseline non-contrast computed tomography (NCCT) scans were performed on admission with a section thickness of 9 mm supratentorially and 4.5 mm infratentorially. ICH hematoma volume was measured using the ABC/2 method, in which *A* is the greatest diameter on the largest hemorrhage slice, *B* is the diameter perpendicular to *A*, and *C* is the approximate number of axial slices with hemorrhage multiplied by the slice thickness (9). uHG was defined as baseline ICH volume (ml) divided by onset to baseline CT time (h) (6). Hematoma locations were classified as lobar, deep (basal ganglia or thalamus), cerebellum, and brainstem. The presence or absence of intraventricular extension (IVE) was also noted. All images were prospectively viewed by a neuroradiologist blinded to clinical data. The neuroradiologists of the study centers were trained centrally with the CT protocol. The missing data were searched from the original medical record.

### Patient Follow-Up and ICH Clinical Outcomes

At 90 days and 1 year after ICH onset, the functional outcomes of all patients were assessed through telephone follow-up interviews to obtain mRS. Every recruited patient with ICH left at least two phone numbers. For patients who were lost to follow-up, we called them once a week for 3 weeks. Telephone follow-up was centralized for all included patients and utilized a standardized interview protocol. The interviewers were trained on the interview protocol.

The primary clinical outcome parameter was the poor outcome at 1 year. Secondary clinical outcomes were mortality in the hospital and poor outcomes at 90 days. Poor outcome was defined as death or major disability (defined by scores 3–6 on the mRS).

### Statistical Analysis

The categorical variables are presented as percentages, and the continuous variables are presented as means and SD if normally distributed or medians and interquartile ranges (IQR) if not normally distributed. Statistical significance for intergroup differences was assessed by Pearson χ^2^ test for categorical variables, and by *t*-test or Wilcoxon test for continuous variables. Multivariable logistic regression analyses were performed to determine the association of uHG speed and baseline hematoma volume with clinical outcomes. The unadjusted logistic regression was not adjusted by covariates. Multivariate logistic regression was adjusted by potential confounding variables according to the results of the univariate analysis. Variables showing *p* < 0.1 in univariate analysis were included in the multivariate model. Key covariates were age, sex, previous anticoagulant therapy, baseline systolic blood pressure (SBP), baseline NIHSS score, ICH location, and IVE. Regression results were reported in odds ratio (OR) and 95% CI. Receiver operating characteristic curves were drawn to estimate possible threshold values of uHG and baseline ICH volume that would optimally predict the primary outcome. The threshold was estimated at the simultaneous maximum sensitivity and specificity. The sensitivity, specificity, positive predictive values (PPV), negative predictive value (NPV), and area under the curve (AUC) of uHG and baseline ICH volume were calculated. All tests were 2-tailed, and *p* < 0.05 was considered statistically significant. All analyses were conducted using SAS version 9.4 statistical software.

## Results

**Figure 1:**
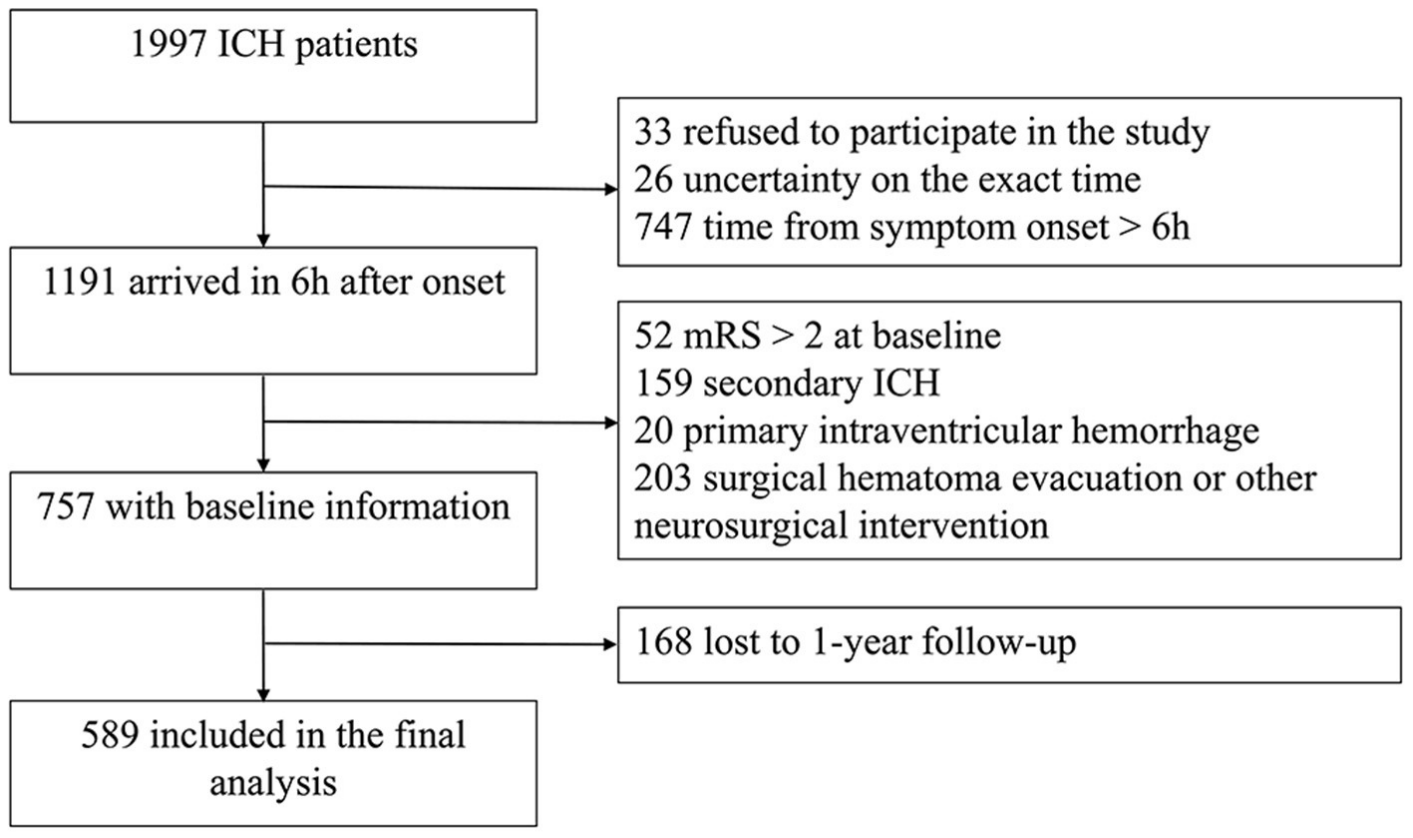
Patients with intracerebral hemorrhage flow chart. ICH, intracerebral hemorrhage; mRS, modified Rankin Scale.

Among 1,997 patients with ICH, 589 patients were included in the present study (Figure 1).

### uHG and the Baseline Characteristics

The median value of uHG was 4.8 (IQR 1.9–12.5) ml/h. The main baseline characteristics of the patients with ICH are summarized in Table 1. The simultaneous maximum sensitivity and specificity for uHG in predicting the primary outcome was 9.3 ml/h. Around 67.9% of patients had uHG ≤ 9.3 ml/h and 32.1% had uHG > 9.3 ml/h. Two patient groups of uHG defined by the cutoff point value were used in the analysis. Patients with faster uHG were more likely to be men, and less likely to have antihypertensive or hypoglycemic therapy. They had higher systolic and diastolic blood pressure, and glucose levels, but lower LDL levels at the baseline. They had higher NIHSS scores, lower GCS scores, and larger hematoma volume at baseline. Patients with ICH with faster uHG presented more frequently in lobar (*p* = 0.001), less frequently in brainstem location (*p* < 0.001), but no significant difference in deep (*p* = 0.509) or cerebellar locations (*p* = 0.076) (Table 1).

**Table 1:** Baseline characteristics of intracerebral hemorrhage (ICH) participants by ultraearly hematoma growth (uHG) levels.

		**uHG**		
	**Total (*n* = 589)**	**≤9.3 ml/h (*n* = 400)**	**>9.3 ml/h (*n* = 189)**	***P*-value**
Age, y, Mean ± SD	58.5 ± 13	58.0 ± 13.0	59.5 ± 13.5	0.763
Male, *n* (%)	378 (64.2)	242 (60.5)	136 (71.9)	0.007
Hypertension history, *n* (%)	408 (69.6)	288 (72.4)	120 (63.8)	0.036
Diabetes history, *n* (%)	98 (16.6)	65 (16.3)	33 (17.5)	0.713
Hyperlipidemia history, *n* (%)	63 (10.7)	43 (10.8)	20 (10.6)	0.951
Antihypertensive therapy, *n* (%)	189 (33.6)	148 (37.0)	50 (26.5)	<0.001
Hypoglycemic therapy, *n* (%)	64 (10.9)	46 (11.5)	18 (9.5)	0.013
Antiplatelet therapy, *n* (%)	86 (14.6)	61 (15.3)	25 (13.2)	0.342
Anticoagulation therapy, *n* (%)	7 (1.2)	6 (1.5)	1 (0.5)	0.053
GCS score, Median (IQR)	14 (9–15)	14 (12–15)	11 (5–14)	<0.001
NIHSS score, Median (IQR)	11 (5–19)	8 (3–14)	17 (11–28)	<0.001
Systolic BP, mmHg, Mean ± SD	171 ± 28.1	168 ± 27.6	176 ± 28.7	0.002
Diastolic BP, mmHg, Mean ± SD	97.3 ± 17.7	96.3 ± 17.4	99.5 ± 18.2	0.024
Platelet count, 10^3^u/L, Mean ± SD	218 ± 64.2	217 ± 61.3	220 ± 70.2	0.438
INR, Mean ± SD	1.3 ± 5.1	1.1 ± 1.5	1.6 ± 8.7	0.566
Glucose, mmol/l, Mean ± SD	7.5 ± 3.2	7.0 ± 2.9	8.5 ± 3.5	<0.001
LDL, mmol/l, Mean ± SD	2.8 ± 0.9	2.9 ± 0.9	2.7 ± 1.1	0.143
Onset to baseline CT time, h, Median (IQR)	2.6 (1.7–4)	3.0 (1.9–4.4)	2.0 (1.1–3.0)	<0.001
ICH volume, ml, Median (IQR)	11.3 (5.1–28.0)	7.6 (3.8–12.5)	39.4 (23.2–67.3)	<0.001
uHG, ml/h, Median (IQR)	4.8 (1.9–12.5)	2.7 (1.4–4.8)	19.0 (12.5–30.9)	<0.001
**ICH location**				<0.001
Lobar, *n* (%)	69 (14.5)	35 (10.3)	34 (24.8)	
Deep, *n* (%)	333 (70.0)	240 (70.8)	93 (67.9)	
Cerebellar, *n* (%)	25 (5.3)	21 (6.2)	4 (2.9)	
Brainstem, *n* (%)	49 (10.3)	43 (12.7)	6 (4.4)	
Intraventricular extension, *n* (%)	50 (8.5)	29 (7.3)	21 (11.1)	0.117
ICH score, Median (IQR)	1 (0.2)	0 (0.1)	2 (1.3)	<0.001
Withdraw, *n* (%)	37 (6.3)	18 (4.5)	19 (10)	0.010

*uHG, ultraearly hematoma growth; SD, standard deviation; IQR, interquartile range; GCS, Glasgow Coma Scale; NIHSS, National Institutes of Health Stroke Scale; BP, blood pressure; INR, international normalized ratio; LDL, low density lipoprotein; ICH, intracerebral hemorrhage*.

**Figure 2:**
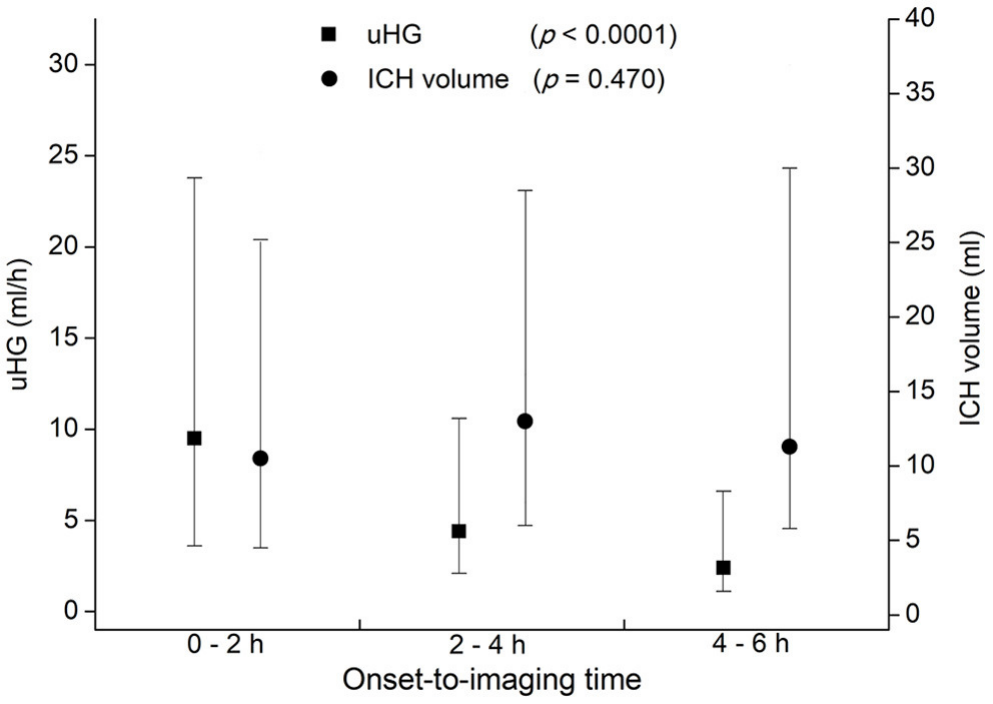
Ultraearly hematoma growth (uHG) and ICH volume by 2-h intervals of time from symptoms onset to baseline CT scan. uHG, ultraearly hematoma growth; ICH, intracerebral hemorrhage; CT, computed tomography.

Patients with ICH and with faster uHG usually arrived at the hospital early. By 2-h intervals from symptoms onset, 207 patients arrived at the hospital in 2 h of OIT. A total of 236 arrived during 2–4 h and 126 arrived during 4–6 h. Patients scanned earlier presented with higher uHG [9.5 (3.6–23.8), 4.4 (2.1–10.6), and 2.4 (1.1–6.6) ml/h, *p* < 0.001]. Three groups of patients had similar ICH volumes [10.5 (4.5–25.2), 13.0 (6.0–28.5), and 11.3 (5.8–30.0) ml, *p* = 0.470] (Figure 2). Other baseline characteristics, such as age, sex, baseline NIHSS score, and baseline GCS score were not associated with onset to baseline CT time.

### uHG, Baseline ICH Volume, and Clinical Outcomes

**Figure 3:**
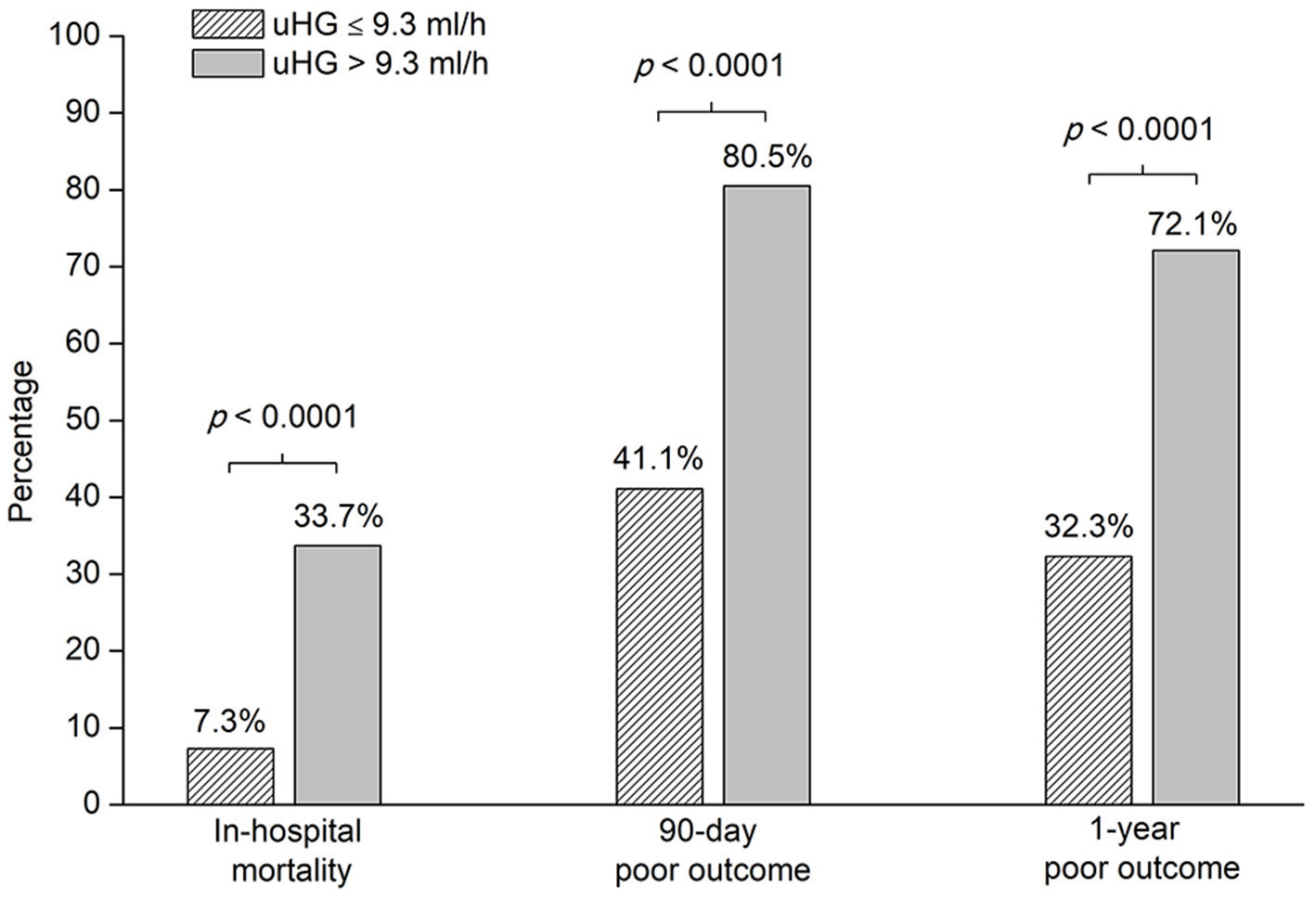
Clinical outcomes of patients with ICH by uHG levels. uHG, ultraearly hematoma growth.

Overall, the proportion of poor outcomes was 45.2% at 1 year after ICH. The mortality was 15.8% in the hospital and the proportion of 90-day poor outcomes was 53.8%. The frequency of in-hospital mortality, 90-day, and 1-year poor outcome after acute ICH was higher in patients with uHG > 9.3 ml/h than in patients with uHG ≤ 9.3 ml/h (Figure 3).

The simultaneous maximum sensitivity and specificity for baseline ICH volume in predicting the primary outcome were 16 ml. Both uHG > 9.3 ml/h and baseline ICH volume > 16 ml were independently related to mortality in hospital, the poor outcome at 90 days, and 1 year in separate multivariate models adjusted for age, sex, anticoagulant use, antiplatelet use, baseline SBP, INR, glucose level, baseline NIHSS, baseline GCS, ICH location, and IVE (Table 2). In multivariate logistic regression analysis, other predictors of in-hospital mortality were age and glucose level. Other predictors of 90-day and 1-year poor outcomes were age, glucose level, and baseline NIHSS (Supplementary Table 1).

**Table 2:** Multivariable logistic regression analyses of uHG and ICH volume on ICH clinical outcomes.

	**uHG** **>** **9.3 ml/h**				**ICH volume** **>** **16 ml**			
	**Unadjusted**		**Adjusted**		**Unadjusted**		**Adjusted**	
	**OR (95% CI)**	***P*-value**	**OR (95% CI)**	***P*-value**	**OR (95% CI)**	***P*-value**	**OR (95% CI)**	***P*-value**
Mortality in hospital	6.54 (4.04–10.6)	<0.001	2.81 (1.52–5.23)	0.001	5.82 (3.54–9.55)	<0.001	2.79 (1.49–5.22)	<0.001
Poor outcome at 90 days	5.85 (3.88–8.82)	<0.001	3.34 (1.87–5.95)	<0.001	5.97 (4.08–8.76)	<0.001	3.76 (2.19–6.47)	<0.001
Poor outcome at 1 year	5.33 (3.64–7.79)	<0.001	3.59 (2.01–6.40)	<0.001	5.12 (3.58–7.35)	<0.001	2.94 (1.73–5.02)	<0.001

*uHG, ultraearly hematoma growth; ICH, intracerebral hemorrhage; OR, odds ratio; CI, confidence interval. Multivariable logistic regression was adjusted by age, sex, anticoagulant use, antiplatelet use, baseline systolic blood pressure, international normalized ratio, glucose level, baseline NIH Stroke score, baseline Glasgow Coma Scale, ICH location, and intraventricular extension*.

The speed of uHG > 9.3 ml/h had higher specificity and PPV but lower sensitivity and NPV than baseline ICH volume >16 ml to predict clinical outcomes, including in-hospital mortality, 90-day, and 1-year poor outcome. Further, uHG > 9.3 ml/h improved the AUC of baseline ICH volume >16 ml in the prediction of in-hospital mortality (Table 3).

**Table 3:** Sensitivity, specificity, predictive values, and area under the curve (AUC) of baseline ICH volume and uHG in the prediction of ICH clinical outcomes.

	**Sensitivity (%)**	**Specificity (%)**	**PPV (%)**	**NPV (%)**	**AUC**
**Mortality in hospital**					
ICH volume >16 ml	73.1	68.1	30.1	93.1	0.791
uHG > 9.3 ml/h	68.8	74.8	33.9	92.8	0.799
**Poor outcome at 90 days**					
ICH volume >16 ml	56.2	82.4	78.8	61.7	0.757
uHG > 9.3 ml/h	48.0	86.4	80.4	58.8	0.729
**Poor outcome at 1 year**					
ICH volume >16 ml	58.6	78.3	69.0	69.7	0.756
uHG > 9.3 ml/h	51.1	83.6	72.0	67.5	0.730

*ICH, intracerebral hemorrhage; uHG, ultraearly hematoma growth; PPV, positive predictive value; NPV, negative predictive value; AUC, area under the curve*.

## Discussion

A higher risk of a poor outcome was seen for the category of faster speed of uHG. The elevated speed of uHG > 9.3 ml/h independently predicts mortality in the hospital, the poor outcome at 90 days, and 1 year. This study also reveals that uHG improved the specificity, not the sensitivity, of the baseline ICH volume in the prediction of clinical outcomes after ICH.

Rodriguez-Luna et al. first analyzed patients with supratentorial ICH and suggested that uHG > 10.2 ml/h was a powerful predictor of HE, early neurologic deterioration (END), and poor outcome at 3 months after acute ICH (6). Based on the datasets of Intensive Blood Pressure Reduction in Acute Cerebral Hemorrhage Trial (INTERACT) (10–12). Sato et al. confirmed that increased hematoma enlargement (HE) and increased poor clinical outcome were observed in patients with ICH of uHG speeds >5 ml/h (7). But these randomized controlled trials included the study population with elevated SBP (150–220 mmHg), which may exclude up to 25% of patients with acute ICH (13). Rodriguez-Luna et al. (8) analyzed the data coming from the Predicting Hematoma Growth and Outcome in Intracerebral Hemorrhage Using Contrast Bolus CT (PREDICT) study (14). Their results suggested that uHG was associated with active bleeding and uHG > 4.7 ml/h was independently related to HE (OR 1.07, 95% CI 1.04–1.11), END, and 3-month poor clinical outcomes (8). However, in the latest single-center study of 137 patients with acute ICH, the OR value for uHG > 4.7 ml/h to predict HE was 0.34 (95% CI 0.12–0.95), which was contrary to a previous study (8, 15). These could in part be explained by differences in the target population, study designs, and low sample size. This study included a heterogeneous ICH patient population coming from routine clinical practice, and the sample size is a big superiority. This study improved the generalizability of uHG in providing early and long-term clinical outcomes after acute ICH.

In China, the percentage of ICH is much higher than that in the western population, and 46% of the patients with ICH have poor outcomes 1 year after onset (16, 17). But the association of uHG with 1-year outcome after acute ICH has not been studied. In this study, 45.2% of the patients with ICH had poor outcomes at 1 year. A 1-year poor outcome was selected as the primary clinical outcome, and the cutoff point value of the speed of uHG was 9.3 ml/h. The risk of 1-year and 90-day poor outcome in the uHG > 9.3 ml/h group was more than 3 times that in the uHG <9.3 ml/h group. Multivariate logistic analysis showed that uHG > 9.3 ml/h was an independent predictor of 1-year poor outcome and 90-day poor outcome. Though in the recent trial, minimally invasive surgery with thrombolysis in intracerebral haemorrhage evacuation (MISTIE III), with the aim of decreasing clot size to 15 mL or less, did not improve the proportion of patients who achieved a good response 365 days after ICH (18). For future trials of patients with ICH who aim to maximize the quality of life at the lowest cost per quality adjusted life year, uHG may be an important marker to identify patients for trial eligibility.

Speed of uHG represents the average bleeding speed before baseline CT scan, although the speed of the HE prior to baseline CT scan cannot be accurately calculated because of the speed changes within the first hours (6). uHG may also be helpful to avoid the possible confounding effect of clinical severity on time to hospital presentation. Patients with faster uHG may have degenerative changes of small vessels, including atherosclerosis, lipohyalinosis, and amyloid angiopathy, to cause larger rupture point, continuous bleeding, rebleeding, and subsequent HG (7, 19–21). In addition, the rapid growth of hematoma may cause more secondary injury surrounding the hematoma (22). Though the same frequency of hypertension history was shown in both slower and faster uHG groups, higher baseline blood pressure and high frequency of lobar hematoma location were shown in the uHG > 9.3 ml/h group. This might represent the causal relationship between high blood pressure and faster uHG, and the causal relationship between amyloid angiopathy and faster uHG for patients with ICH without hypertension history.

Accurate selection of patients with ICH with poor prognosis is a key factor for emergency treatment to improve prognoses, such as aggressive blood pressure lowing therapy, hemostatic treatment, and minimally invasive catheter evacuation in ICH. In our study, the risk of in-hospital mortality in the uHG > 9.3 ml/h group was 2.81 times that in the uHG <9.3 ml/h group. The OR value of uHG decreased in adjusted multivariate logistic regression analysis because age and glucose level were additional independent predictors of ICH clinical outcomes, which have been previously reported (3, 16, 23, 24). This seemed to exert a relevant confounding effect on the predicted value of uHG. But after adjusting for these factors, uHG > 9.3 ml/h was an independent predictor of in-hospital mortality and improved the AUC of baseline ICH volume > 16 ml to predict mortality in hospital. uHG is expected to be a more specific predictor for a higher risk of in-hospital death. This helps to specifically identify patients with ICH who may benefit the most from early, aggressive monitoring, and treatment. This hypothesis would need to be tested in a randomized clinical trial that delivers an aggressive intervention(s) compared with standard medical care.

Patients with ICH who arrive at the hospital earlier may have a faster uHG despite having a similar hematoma volume, especially the patients who arrive at the hospital within 2 h of onset. This is consistent with other studies (8). It had been reported that an early HG has been demonstrated only 1 h after a baseline CT scan performed during the first hours and uHG may act as a surrogate of the speed of HE (6, 25). This finding has potential implications for future clinical trials design, which can accurately select patients with ICH who may get more benefit from hemostatic or aggressive lowering blood pressure treatment if treated in ultraearly time window, for example, within 2 h from symptoms onset.

Mortality in hospital, the poor outcome at 90 days, and 1 year were related to both uHG and baseline ICH volume, without interaction. Being different from what had been reported in previous studies (6–8), uHG > 9.3 ml/h improved the specificity and PPV, but not the sensitivity and NPV of the baseline ICH volume > 16 ml in the prediction of all these clinical outcomes. Therefore, the combination of both uHG and the baseline ICH volume could allow better stratification of ICH patients at high risk of early and long-term poor clinical outcomes.

There were some limitations in this study. First, 168 patients missed 1-year follow-up. To mitigate this limitation, we analyzed the characteristics of these patients compared with patients who had complete follow-up. Patients who lost to 1-year follow-up were younger (54.4 ± 13.1 vs. 58.5 ± 13, *p* < 0.001) and had lower rate of antiplatelet therapy (7.8 vs. 14.6%, *p* = 0.008). The other baseline characteristics did not have a significant difference between the two groups, such as baseline NIHSS score, baseline blood pressure, hematoma volume, hemorrhage location, IVE, and speed of uHG (Supplementary Table 2). Second, as demonstrated in previous studies, uHG was a powerful predictor of HE, but our work was not designed to explore the relationship between uHG and HE.

## Conclusions

This study, with a larger study population with a 1-year follow-up published to date, provides additional evidence that uHG is a useful predictor of death in hospitals and poor outcome at 90 days and 1 year after ICH. Stratification of the ICH patients based on the uHG combined with baseline hematoma volume is helpful for physicians to identify patients who need more aggressive treatments and is useful for future selection of patients for clinical trials to improve early and long-term clinical outcomes.

## Data Availability Statement

The raw data supporting the conclusions of this article will be made available by the authors, without undue reservation.

## Ethics Statement

The studies involving human participants were reviewed and approved by the Institutional Review Board (IRB) of the Beijing Tiantan Hospital Affiliated to Capital Medical University. The patients/participants provided their written informed consent to participate in this study.

## Author Contributions

W-JW conceived of the study, performed the statistical analyses, and drafted the manuscript. J-JL and L-PL helped draft the manuscript. J-KJ participated in data collection. X-QZ conceived of the study, participated in coordination, and helped draft the manuscript. All authors contributed to the article and approved the submitted version.

## Funding

This study was sponsored by Capital health research and development of special (grant umber: Capital 2011-2004-03; http://wjw.beijing.gov.cn/; institute: Beijing Municipal Commission of Health and Family Planning; the author who received the funding: X-QZ) and Beijing Municipal Science & Technology Commission, PR China (grant number: Z131107002213009; http://www.bjkw.gov.cn/; the author who received the funding: X-QZ). The funders had no role in study design, data collection and analysis, decision to publish, or preparation of the manuscript.

## Conflict of Interest

The authors declare that the research was conducted in the absence of any commercial or financial relationships that could be construed as a potential conflict of interest.

## Publisher's Note

All claims expressed in this article are solely those of the authors and do not necessarily represent those of their affiliated organizations, or those of the publisher, the editors and the reviewers. Any product that may be evaluated in this article, or claim that may be made by its manufacturer, is not guaranteed or endorsed by the publisher.
