# Supplementary material for: Ultraearly Hematoma Growth in Acute Spontaneous Intracerebral Hemorrhage Predicts Early and Long-Term Poor Clinical Outcomes: A Prospective, Observational Cohort Study
Source: Front Neurol. 2021 Dec 15;12:747551. doi: 10.3389/fneur.2021.747551 (PMC8714734; doi:10.3389/fneur.2021.747551)
Supplement: Supplementary file 1 [file Table_1.DOCX]

**Supporting information**

**Table S1. Multivariable logistic regression analyses of other predictors on intracerebral hemorrhage clinical outcomes.**

|  | **in-hospital mortality** | | **90-day poor outcome** | | **1-year poor outcome** | |
| --- | --- | --- | --- | --- | --- | --- |
|  | OR (95% CI) | *p* Value | OR (95% CI) | *p* Value | OR (95% CI) | *p* Value |
| **age** | 1.03 (1.01 - 1.05) | 0.045 | 1.06 (1.04 - 1.09) | < 0.001 | 1.09 (1.07 - 1.12) | < 0.001 |
| **glucose level** | 1.21 (1.11 - 1.32) | < 0.001 | 1.13 (1.03 - 1.23) | < 0.001 | 1.17 (1.08 - 1.29) | < 0.001 |
| **baseline NIHSS** | / | / | 1.28 (1.21 - 1.35) | < 0.001 | 1.22 (1.15 - 1.29) | < 0.001 |

OR = odds ratio; CI = confidence interval; NIHSS = NIH Stroke Scale. Multivariable logistic regression was adjusted by age, sex, anticoagulant use, antiplatelet use, baseline systolic blood pressure, international normalized ratio, glucose level, baseline NIH Stroke score, baseline Glasgow Coma Scale, ICH location and intraventricular extension.
