# Supplementary material for: Ultraearly Hematoma Growth in Acute Spontaneous Intracerebral Hemorrhage Predicts Early and Long-Term Poor Clinical Outcomes: A Prospective, Observational Cohort Study
Source: Front Neurol. 2021 Dec 15;12:747551. doi: 10.3389/fneur.2021.747551 (PMC8714734; doi:10.3389/fneur.2021.747551)
Supplement: Supplementary file 2 [file Table_2.DOCX]

**Supporting information**

**Table S2. Baseline characteristics of intracerebral hemorrhage (ICH) participants 1-year follow up and lost to 1-year follow up.**

|  |  | **1-year follow up** | |  |
| --- | --- | --- | --- | --- |
|  | Total (n=757) | Lost to follow up (n=168) | Follow up (n=589) | *p* Value |
| **Age, y, Mean ± SD** | 57.6 ± 13.3 | 54.4 ± 13.1 | 58.5 ± 13 | < 0.001 |
| **Male, n (%)** | 496 (65.5) | 118 (70.2) | 378 (64.2) | 0.145 |
| **Hypertension history, n (%)** | 533 (70.9) | 125 (75.3) | 408 (69.6) | 0.155 |
| **Diabetes history, n (%)** | 120 (15.9) | 22 (13.1) | 98 (16.6) | 0.267 |
| **Hyperlipidemia history, n (%)** | 80 (10.6) | 17 (10.1) | 63 (10.7) | 0.830 |
| **Antihypertensive therapy, n (%)** | 233 (36.9) | 35 (27.8) | 189 (33.6) | 0.174 |
| **Hypoglycemic therapy, n (%)** | 73 (10.6) | 9 (6.3) | 64 (10.9) | 0.058 |
| **Antiplatelet therapy, n (%)** | 97 (14.8) | 25 (14.9) | 72 (12.2) | 0.089 |
| **Anticoagulation therapy, n (%)** | 9 (1.3) | 2 (1.4) | 7 (1.2) | 0.931 |
| **GCS score, Median (IQR)** | 14 (9 - 15) | 14 (8 - 15) | 14 (9 - 15) | 0.817 |
| **NIHSS score, Median (IQR)** | 11 (5 - 19) | 11 (5 - 19) | 11 (5 - 19) | 0.817 |
| **Systolic BP, mmHg , Mean ± SD** | 170 ± 28.0 | 170 ± 29.0 | 171 ± 28.1 | 0.435 |
| **Diastolic BP, mmHg, Mean ± SD** | 98 ± 18.0 | 100 ± 19.0 | 97.3 ± 17.7 | 0.278 |
| **Platelet count, 10^3^u/L, Mean ± SD** | 218 ± 62.9 | 220 ± 58.0 | 218 ± 64.2 | 0.750 |
| **INR, Mean ± SD** | 1.2 ± 4.5 | 1.0 ± 0.2 | 1.3 ± 5.1 | 0.402 |
| **Glucose, mmol/l, Mean ± SD** | 9.0 ± 3.7 | 14.3 ± 3.4 | 7.5 ± 3.2 | 0.368 |
| **Onset to baseline CT time, h, Median (IQR)** | 2.7 (1.7 - 4.2) | 2.9 (1.9 - 4.5) | 2.6 (1.7 - 4) | 0.048 |
| **ICH volume, ml, Median (IQR)** | 11.6 (5.3 - 27.6) | 12.8 (5.3 - 26.6) | 11.3 (5.1 - 28.0) | 0.937 |
| **uHG, ml/h, Median (IQR)** | 4.9 (1.9 - 11.8) | 5.1 (1.7 - 11.2) | 4.8 (1.9 - 12.5) | 0.419 |
| **ICH location** |  |  |  | 0.108 |
| **lobar, n (%)** | 80 (13.4) | 11 (9.0) | 69 (14.5) |  |
| **Deep, n (%)** | 433 (72.4) | 100 (82.0) | 333 (70.0) |  |
| **Cerebellar, n (%)** | 26 (4.4) | 1 (0.8) | 25 (5.3) |  |
| **Brainstem, n (%)** | 59 (9.9) | 10 (8.2) | 49 (10.3) |  |
| **Intraventricular extension, n (%)** | 57 (7.53) | 7 (4.2) | 50 (8.5) | 0.061 |

Abbreviations: uHG = ultraearly hematoma growth; SD = standard deviation; IQR = interquartile range; GCS = Glasgow Coma Scale; NIHSS = NIH Stroke Scale; BP = blood pressure; INR = international normalized ratio; ICH = intracerebral hemorrhage.
